# Control of a Robotic Arm With an Optimized Common Template-Based CCA Method for SSVEP-Based BCI

**DOI:** 10.3389/fnbot.2022.855825

**Published:** 2022-03-15

**Authors:** Fang Peng, Ming Li, Su-na Zhao, Qinyi Xu, Jiajun Xu, Haozhen Wu

**Affiliations:** ^1^Zhongshan Institute, University of Electronic Science and Technology of China, Zhongshan, China; ^2^School of Automation Engineering, University of Electronic Science and Technology of China, Chengdu, China; ^3^College of Electrical and Information Engineering, Zhengzhou University of Light Industry, Zhengzhou, China; ^4^School of Automation, Guangdong University of Technology, Guangzhou, China

**Keywords:** brain-computer interface (BCI), steady-state visual evoked potential (SSVEP), robotic arm, optimized common template based canonical correlation analysis (OCT-CCA), spatial filter

## Abstract

Recently, the robotic arm control system based on a brain-computer interface (BCI) has been employed to help the disabilities to improve their interaction abilities without body movement. However, it's the main challenge to implement the desired task by a robotic arm in a three-dimensional (3D) space because of the instability of electroencephalogram (EEG) signals and the interference by the spontaneous EEG activities. Moreover, the free motion control of a manipulator in 3D space is a complicated operation that requires more output commands and higher accuracy for brain activity recognition. Based on the above, a steady-state visual evoked potential (SSVEP)-based synchronous BCI system with six stimulus targets was designed to realize the motion control function of the seven degrees of freedom (7-DOF) robotic arm. Meanwhile, a novel template-based method, which builds the optimized common templates (OCTs) from various subjects and learns spatial filters from the common templates and the multichannel EEG signal, was applied to enhance the SSVEP recognition accuracy, called OCT-based canonical correlation analysis (OCT-CCA). The comparison results of offline experimental based on a public benchmark dataset indicated that the proposed OCT-CCA method achieved significant improvement of detection accuracy in contrast to CCA and individual template-based CCA (IT-CCA), especially using a short data length. In the end, online experiments with five healthy subjects were implemented for achieving the manipulator real-time control system. The results showed that all five subjects can accomplish the tasks of controlling the manipulator to reach the designated position in the 3D space independently.

## 1. Introduction

How to realize the information interaction between people and external equipment simply and conveniently has always been the goal of human beings, and the brain-computer interface (BCI) provides this possibility. Specifically, BCI is a control and communication system that can recognize or convert brain activity information into control commands of external devices (Wang et al., 2020). Compared to ordinary input interactive devices, the BCI input is the brain signals recorded by electrodes on the head, and the output applications can be controlled directly from the brain, such as robotic arms (Aljalal et al., 2020; Zhu et al., 2020), wheelchairs (Li et al., 2016; Deng et al., 2019; Bonci et al., 2021), character speller systems (Rezeika et al., 2018; Podmore et al., 2019), and other devices (Gao et al., 2019). Nowadays, many researchers intended to use BCI to develop an assistant for people who suffer from severe neuromuscular disorders, which can help these people with disabilities can control robotic arms directly by analyzing their brain activities without body movement (Meng et al., 2016; Chen et al., 2018, 2020; Bonci et al., 2021).

Nowadays, a robotic arm can be controlled *via* measuring and recording electroencephalogram (EEG) signals, which is progressively used in BCI applications of non-invasive modalities for its practicality and security (Gao et al., 2003; Kumar and Reddy, 2020). Several commonly used EEG paradigms include steady-state visual evoked potential (SSVEP), P300 (Farwell et al., 2014; Yin et al., 2016), and motor imagery (MI) (Song and Kim, 2019; Xu et al., 2021). Compared to the EEG paradigms of P300 and MI, the SSVEP-based BCI system is preferable in robotic arms control owing to the little training and relatively high recognition accuracy (Ge et al., 2019; Chen et al., 2020; Zhang et al., 2020). Besides, due to the limited output commands, it is not able to perform a real-time motion control task in a 3D space for the P300-based and MI-based robotic arm systems with multiple degrees of freedom (DOF) (Xu et al., 2019). Therefore, this study focuses on the SSVEP-based robotic arm control system to provide multiple command options, the subjects can elicit the evoked potentials to obtain EEG signals by gazing at visual flickers and the commands of the 3D motion of the robotic arm are generated from the result of the SSVEP signals recognition. It can be accepted that how to improve the SSVEP recognition accuracy is a key factor in determining the performance of the entire SSVEP-based robotic arm system.

So far, numerous novel and improved approaches have been proposed for SSVEP recognition and classification. A conventional and simple way is to use the power spectrum density analysis (PSDA) in the frequency domain (Hwang et al., 2012), such as the fast Fourier transform (FFT) finds the magnitude of EEG signals at each stimulus frequency for target detecting (Chen et al., 2017). However, the inapplicability of PSDA to multi-channel and real-time BCI systems is now being replaced by another simple and practical method, namely the canonical correlation analysis (CCA) method (Nakanishi et al., 2017). Compared to the PSDA method, the CCA method obtained a better signal-to-noise ratio (SNR) since it can utilize multi-channel data and higher harmonic frequencies of SSVEP related components in EEG signals (Bin et al., 2011; Hakvoort et al., 2011). At the same time, some improved methods for the deficiencies of the CCA method have also been proposed. Among them, the multi-way CCA (MCCA) and L1-regularized MCCA improve recognition performance by optimizing reference signals (Zhang et al., 2011, 2013). The filter bank CCA (FBCCA) method enhances SSVEP detection by decomposing the signal into sub-band and further using the harmonic information in them (Chen et al., 2015). Furthermore, there are approaches such as individual template-based CCA (IT-CCA) and transfer template-based (tt-CCA) that used real EEG data to construct new signal templates for frequency identification (Nakanishi et al., 2015; Yuan et al., 2015; Wang et al., 2020). Many comparative studies showed that the extension methods of CCA with supervised (such as IT-CCA) have better performance of recognition accuracies and information transfer rate (ITR) than training-free (such as CCA and FBCCA) (Nakanishi et al., 2015; Zerafa et al., 2018; Saidi et al., 2019). However, for the IT-CCA method, the individual templates only extract the frequency and phase information from individual data, and the final spatial filters are obtained online from the calibration data and EEG signals, which could cause overfitting owing to the short of common features from various subjects. On the other hand, the reference signals of sine-cosine only consider an ideal frequency template, which may not be optimum for frequency recognition owing to containing no real SSVEP features. Hence, this study built the optimized common templates (OCTs) from various subjects to broaden its applicability and enhance the SNR of SSVEP.

Inspired by the above methods and their potential problems, first, this study introduces a novel extended template-based method with supervised, which aims at finding more efficient OCTs from various subjects. Subsequently, the spatial filters are formulated from the common templates and the EEG signals to improve the SNR and extract the SSVEP-related component that can result in a better performance. Furthermore, an SSVEP-based seven-DOF robotic arm control system with six output commands or stimulus targets is designed to validate the performance. In the performance evaluation, a comparison offline experiment and a real-time control online experiment were designed to explore the performance of the OCT-CCA method and the completion of the robotic arm control system. The results of the offline and online experiment demonstrate that the OCT-CCA approach have a superiority of frequency recognition accuracy than CCA and IT-CCA method, and the subjects in robotic arm control can complete the assignment of controlling a robotic arm to reach a specified position in 3D space by 6 kinds of stimulus frequencies or commands in SSVEP-based BCI.

## 2. Materials and Methods

### 2.1. System Description

**Figure 1:**
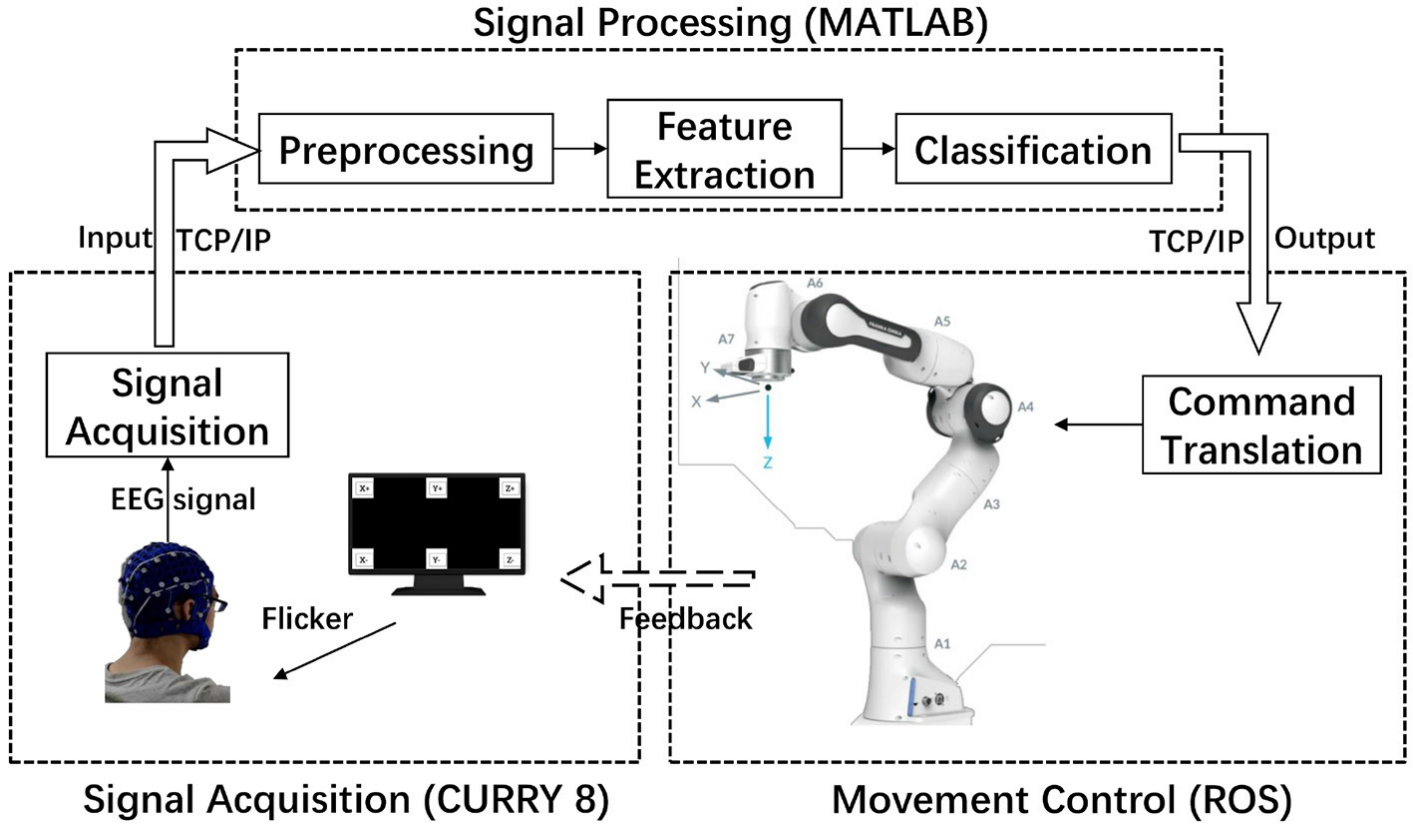
Overview of the proposed steady-state visual evoked potential (SSVEP)-based robotic arm control system.

**Figure 2:**
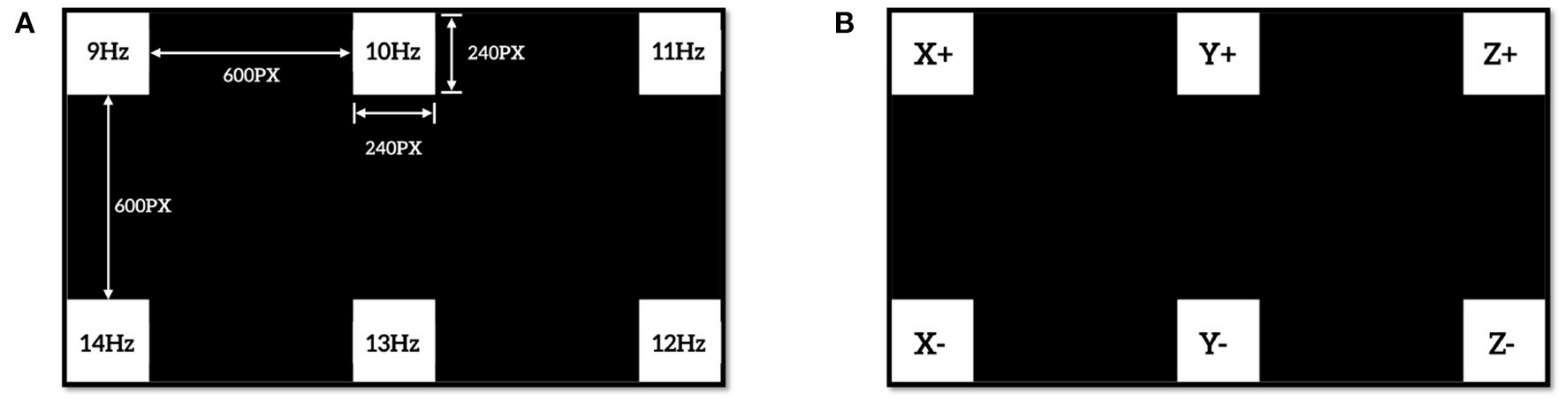
Visual stimulus layout of SSVEP experiment, **(A)** is the layout and frequency values of SSVEP stimulation box with six targets, **(B)** is a command matrix for the robotic arm control.

The schematic architecture of the introduced robotic arm control system is shown in Figure 1, which can be divided into three stages: signal acquisition, signal processing, and movement control of the robotic arm. First, the EEG signals are gathered by the specific electrodes using an amplifier (Grael EEG V2, Compumedics, Inc.) and sent to the signal processing stage. Second, the EEG signals are processed by signal preprocessing, feature extraction, and classification to generate classification results. At this stage, the classification result is provided by extracting and analyzing the features in the EEG signal, and then the classification result is sent to the next stage for robotic arm control. A 7-DOF robotic arm (Franka Emika Panda) with an 855 mm working radius was used to perform specified operation tasks in this study, which can be programmed by C++ and Robot Operating System (ROS). In the final movement control stage, according to the correspondence between the stimulus frequencies and the motion commands, the classification results are converted into motion control commands of the robotic arm and received by the robotic arm controller, and the results will be displayed on the screen as feedback to the subject at the same time. Figure 2 shows the commands of the robotic arm and their corresponding stimulation frequencies. The six commands here represent the six directions in which participants can manipulate the robotic arm to perform the desired motion in 3D space. Finally, the robotic arm executes the corresponding actions in the 3D space actuated by the motion command, and then the subject generates the next command based on the observed error between the actual position of the robotic arm and the expected position. The signals were recorded with the Data Acquisition software from the CURRY 8 (Compumedics, Inc.), the data analysis program and the stimulation program were developed under MATLAB (Mathworks, Inc.) (Nakanishi et al., 2014a; Gao et al., 2019). The experiment was conducted with the consent of each subject.

### 2.2. Target Identification Algorithm

#### 2.2.1. Standard CCA for SSVEP Recognition

In the CCA method, the spatial filters are gained to maximize the correlation coefficient between the two multivariate variables (Hardoon et al., 2004; Bin et al., 2011). For SSVEP recognition, the EEG signals $X\in {\mathbb{R}}^{N_{c}\times N_{p}}$ is given from *N*_*c*_ channels, where *N*_*p*_ is the number of sampling points. When the frequency is *f*_*k*_ (*k* = 1, 2, ...*K*), the reference signals $Y_{k}\in {\mathbb{R}}^{2N_{h}\times N_{p}}$ can be pre-constructed as follows:


(1) $$\begin{array}{l} Y_{k}=\left[\begin{array}{c} sin\left(2\pi f_{k}n\right)\\ cos\left(2\pi f_{k}n\right)\\ \vdots \\ sin\left(2\pi N_{h}f_{k}n\right)\\ cos\left(2\pi N_{h}f_{k}n\right) \end{array}\right]\;n=\frac{1}{F_{s}},\frac{2}{F_{s}},...\frac{N_{p}}{F_{s}} \end{array}$$

where *N*_*h*_ denotes the number of harmonics, *F*_*s*_ refers to the sampling rate, and *n* can be regarded as a time series in a sine or cosine wave, which has the same length as EEG signals *X*. Specifically, the CCA method finds the projection vectors $w_{x}\in {\mathbb{R}}^{N_{c}}$ and $w_{y}\in {\mathbb{R}}^{N_{h}}$ to maximize the correlation coefficient ρ_*k*_ between the linear combinations $w_{x}^{T}X$ and $w_{y}^{T}Y_{k}$ by solving the problem shown in Equation (2).


(2) $$\begin{array}{l} \underset{w_{x},w_{y}}{max}\rho_{k}=\frac{E\left[w_{x}^{T}XY_{k}^{T}w_{y}\right]}{\sqrt{E\left[w_{x}^{T}XX^{T}w_{x}\right]E\left[w_{y}Y_{k}Y_{k}^{T}w_{y}\right]}} \end{array}$$

Here, the optimization problem described above can be solved by the Lagrange multiplier method (Friman et al., 2001). As shown in Equation (3), the target frequency *f*_*target*_ of the SSVEP is recognized by the largest feature ρ_*k*_.


(3) $$\begin{array}{l} f_{target}=\underset{f_{k}}{argmax}\;\rho_{k},\;k=1,2,...,K \end{array}$$

#### 2.2.2. IT-Based CCA for SSVEP Recognition

The individual template-based CCA method built individual templates *M*_*k*_ (*k* = 1, 2, ...*K*) by averaging multiple training trials to utilize the subject individual information and enhance the SNR of SSVEP (Nakanishi et al., 2015; Zerafa et al., 2018). Similar to the CCA method, IT-CCA is also used to find the maximum correlation between the two multivariate variables, but the reference signal *Y*_*k*_ is replaced by the individual template *M*_*k*_. The spatial filter *w*_*m*_ has the same function as the *w*_*y*_ in Equation (2), which aims to maximize the correlation coefficient ρ_*k*_. More specifically, the correlation coefficient ρ_*k*_ can be obtained as follows:


(4) $$\begin{array}{l} \underset{w_{x},w_{m}}{max}\rho_{k}=\frac{w_{x}^{T}XM_{k}^{T}w_{m}}{\sqrt{w_{x}^{T}XX^{T}w_{x}\;\cdot \;w_{m}M_{k}M_{k}^{T}w_{m}}} \end{array}$$

#### 2.2.3. OCT-Based CCA Method for SSVEP Recognition

Although many research studies have shown the superiority of SSVEP detection of the standard CCA and IT-CCA method, the reference signals of sine-cosine only consider an ideal frequency template, which includes no abundant SSVEP related components features from recorded training data (Nakanishi et al., 2016; Wang et al., 2020). Besides, the subject-specific training method like IT-CCA hardly achieves optimal recognition accuracy since the EEG signals are easily disturbed by spontaneous EEG or noise (Wong et al., 2020). To overcome the above difficulties, one possible way is to build more efficient templates for CCA and IT-CCA methods.

Assume $X_{k,t}\in {\mathbb{R}}^{N_{c}\times N_{p}}$ is the *t*-th calibration trail's SSVEP data of visual stimulus frequency *f*_*k*_ (*k* = 1, 2, ..., *K*). The subject's SSVEP template ${\;}^{n}{\bar{X}}_{k}\in {\mathbb{R}}^{N_{c}\times N_{p}}$ obtained by averaging training trials from the same subject as ${\;}^{n}{\bar{X}}_{k}=\frac{1}{N_{t}}\overset{N_{t}}{\underset{t=1}{\sum }}X_{k,t}\;\left(n=1,2,...,N\right)$, where *n* indicates the index of subjects, and *N*_*t*_ denotes the number of training trials. Since there are multi-stimulus targets for each subject, the inter-subject SSVEP templates are defined as


(5) X¯k=[X¯1 kT,X¯2 kT,...,X¯N kT]T, k=1,2,...,K,

where ${\bar{X}}_{k}\sim N\left(\mu ,\Sigma \right)$, $\mu =E\left[{\bar{X}}_{k}\right]\in {\mathbb{R}}^{N}$ is a sample mean vector, and $\Sigma =E\left[{\left({\bar{X}}_{k}-\mu \right)}^{2}\right]\in {\mathbb{R}}^{N\times N}$ is a sample covariance matrix (where *E*[·] denotes expectation). To capture the common information and increasing the robustness for target recognition among the inter-subject SSVEP templates, a set of vectors $\omega_{n}\in {\mathbb{R}}^{N_{c}\times 1}\;\left(n=1,2,...,N\right)$ were defined to optimize the templates ${\bar{X}}_{k}$. Then a transformation is implemented for the SSVEP templates ${\bar{X}}_{k}$, and the OCTs were defined as


(6) ωTX¯k=[X¯1 kTw1,X¯2 kTw2,⋯,X¯N kTwN]T              =[X^1 kT,X^2 kT,...,X^N kT]T=X^k, k=1,2,...,K,

where ${\;}^{n}{\hat{X}}_{k}={\omega_{n}^{T}}^{n}{\bar{X}}_{k}\in {\mathbb{R}}^{1\times N_{p}}\;\left(n=1,2,...N\right)$, and ${\hat{X}}_{k}\in {\mathbb{R}}^{N\times N_{p}}$ denotes the optimized common template at frequency *f*_*k*_. The projection vectors ω_*n*_(*n* = 1, 2, ..., *N*) are used to optimize characteristics of the SSVEP templates ${\bar{X}}_{k}$ to obtain the high correlations between the new OCTs ${\;}^{n_{1}}{\hat{X}}_{k}$ and ${\;}^{n_{2}}{\hat{X}}_{k}$.

Without loss of generality, the signals are normalized to zero mean and the sample covariance matrix for signal *X* can be calculated by *K* = *XX*^*T*^. Of course, E[X¯nk]=0. We can get ${\hat{X}}_{k}\sim N\left(\hat{\mu },\hat{\Sigma }\right)$, where $\hat{\Sigma }=\left\{{\hat{\Sigma }}_{n_{1}n_{2}}\right\}\in {\mathbb{R}}^{N\times N}$ is a covariance matrix. To explore the overall correlation with the real SSVEP data among the multiple subjects, then all possible correlations can be described as


(7) ∑n1,n2=1NΣ^n1n2=∑n1,n2=1NE[(ωn1T· X¯n1 k−E[ωn1T· X¯n1 k])                           (X¯n2 kT·ωn2−E[n2X¯kT·ωn2)]                     =Var(∑n1=1Nωn1TX¯n1 k)                     =∑n1=1N∑n2=1Nωn1TΣn1n2ωn2

where *Var*{·} denote the variance of the random vectors. Also, a correlation matrix *C* is defined as $C_{ij}=Corr{(}^{i}{\hat{X}}_{k}{,}^{j}{\hat{X}}_{k})$ to quantify new variables, where the principal diagonal of the correlation matrix *C* is the correlation of a random variable with itself, which leads to


(8) $$\begin{array}{l} Corr\left({\omega_{n}^{T}}^{n}{\bar{X}}_{k}{,}^{n}{\bar{X}}_{k}^{T}\omega_{n}\right)=1,n=1,2,...N \end{array}$$

In this case, we optimized Equation (7) to maximize all covariances between the new variables simultaneously, and the optimization function can be converted as Equation (9) by using the Lagrangian multiplier method under the constraint in Equation (8).


(9) $$\begin{array}{ll} J & =\overset{N}{\underset{n_{1}=1}{\sum }}\overset{N}{\underset{n_{2}=1}{\sum }}\omega_{n_{1}}^{T}\Sigma_{n_{1}n_{2}}\omega_{n_{2}}-\overset{N}{\underset{n_{1}=1}{\sum }}\lambda \left(\omega_{n_{1}}^{T}\Sigma_{n_{1}n_{1}}\omega_{n_{1}}-1\right) \end{array}$$

**Figure 3:**
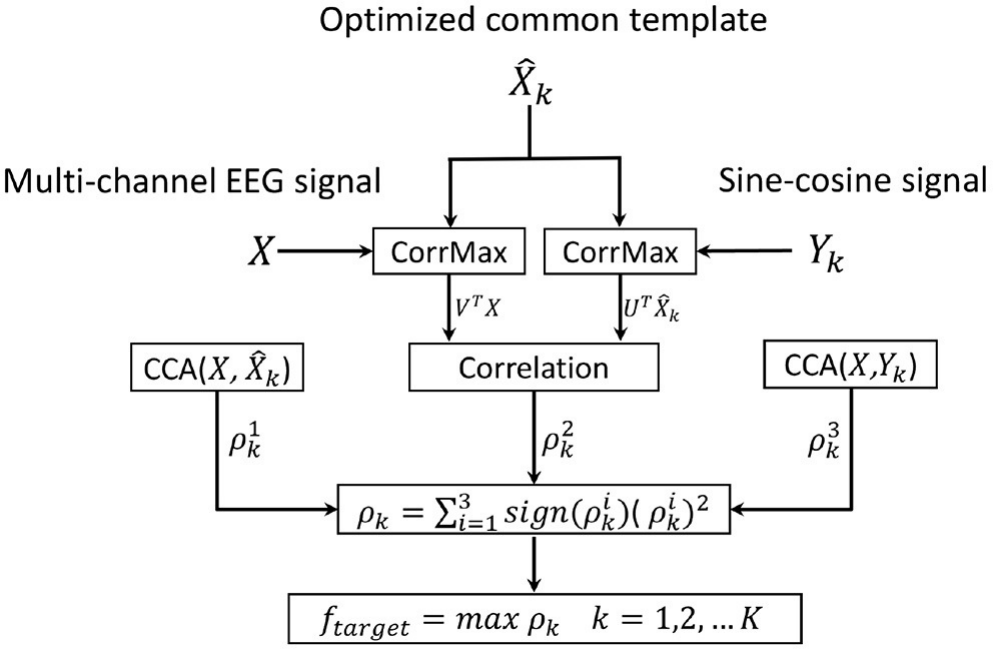
Flowchart of the optimized common template-based canonical correlation analysis (OCT-CCA) method for SSVEP frequency recognition.

To maximize Equation (9), we can set the first dervivate ∂*J*(ω_*n*_1__)/∂ω_*n*_1__ = 0, and get


(10) $$\begin{array}{l} \overset{N}{\underset{\begin{array}{c} n_{2}=1 \end{array}}{\sum }}\Sigma_{n_{1}n_{2}}\omega_{n_{2}}=\lambda \Sigma_{n_{1}n_{1}}\omega_{n_{1}},\;n_{1}=1,2,...N \end{array}$$

where Equation (10) is a symmetric generalized eigensystem, and we can obtain the desired projection vectors for by computing the eigenvectors ω_*n*_(*n* = 1, 2, ...*N*) corresponding to the eigenvalues λ_1_ ≥ λ_2_ ≥ ⋯ ≥ λ_*N*_ of the above generalized eigenvalue problem, and the OCT can be computed by (6). Figure 3 illustrates the OCT based CCA method for SSVEP recognition.

After obtaining the OCTs ${\hat{X}}_{k}\;\left(k=1,2,...,K\right)$, two spatial filters *U* and *V* are computed for each template ${\hat{X}}_{k}$ at frequency *f*_*k*_, the former is used to retain the SSVEP-related components in the OCTs with the stimulation frequency and the latter is used to utilize the subject's individual information. Formally, the spatial filter *U* is calculated by maximizing the similarity between the OCTs ${\hat{X}}_{k}$ and the reference signal *Y*_*k*_. To this end, we can use the covariance $Cov\left(U^{T}{\hat{X}}_{k},W^{T}Y_{k}\right)$ to calculate the maximal correlation between $U^{T}{\hat{X}}_{k}$ and $W^{T}Y_{k}$. Similar to the CCA method, the spatial filter *U* can be gained by transforming the optimization function in Equation (11) into the generalized eigenvalue problem in Equation (12) (Borga, 1998; Sun et al., 2010).


(11) $$\begin{array}{l} \underset{U}{max}\;tr\left(U^{T}{\hat{X}}_{k}Y_{k}^{T}W\right)\\ s.t.U^{T}{\hat{X}}_{k}Y_{k}^{T}W=\Lambda \\ \;U^{T}{\hat{X}}_{k}{\hat{X}}_{k}^{T}U=W^{T}Y_{k}Y_{k}^{T}W=I, \end{array}$$

where Λ is a diagonal matrix, and *I* is the identity matrix. In that way, the corresponding generalized eigenvalue problem is


(12) $$\begin{array}{l} {\hat{X}}_{k}Y_{k}^{T}{\left(Y_{k}Y_{k}^{T}\right)}^{-1}Y_{k}{\hat{X}}_{k}^{T}U=\Lambda {\hat{X}}_{k}{\hat{X}}_{k}^{T}U \end{array}$$

Hence, the diagonal matrix Λ is made up of the eigenvalue corresponding to the eigenvector, and the spatial filter *U* can be found by solving equation (12). Equivalently, we can use the same method as above to calculate the spatial filter *V* between the EEG signal *X* and the OCTs ${\hat{X}}_{k}$.

Based on the two spatial filters obtained above, the linear combination $U^{T}{\hat{X}}_{k}$ and *V*^*T*^*X* is exploited for SSVEP recognition by calculating Pearson correlation coefficient (PCC) as shown in Figure 3. In addition, for each stimulus frequency, the CCA method was also adopted to maximize the correlation between the EEG signal *X* and the OCT ${\hat{X}}_{k}$, and the correlation between the EEG signal *X* and the reference signal *Y*_*k*_. Finally, the three correlation coefficients described above are utilized to combine as the recognition feature for classification:


(13) $$\begin{array}{l} \rho_{k}=\overset{3}{\underset{i=1}{\sum }}sign\left(\rho_{i}\right)\rho_{i}^{2}. \end{array}$$

The stimulation frequency *f*_*k*_ can be identified by Equation (3).

### 2.3. Datasets Acquisition and Preprossing

Five healthy volunteers (mean age 21 years) participated in the online experiments. Many studies have shown that 8–15 Hz is the optimal range of stimulation frequencies for the evoked signal to obtain a higher SNR and amplitude (Nakanishi et al., 2014b; Wang et al., 2017). In this study, six white visual stimulus squares flicker at 9 Hz, 10 Hz, 11 Hz, 12 Hz, 13 Hz, and 14 Hz on an LCD monitor with a refresh rate of 60 Hz and a screen resolution of 1,920 × 1,080. Each stimulus target is a square with size 240 × 240 pixels, and the distance between two neighboring targets was 600 pixels, as shown in Figure 2A. The amplifier records the raw EEG signals at a sampling rate of 1,024 Hz and eventually is down-sampled to 256 Hz to reduce the cost of real-time calculation. To collect higher quality signals with lower impedance and increase the spatial resolution of EEG, digital filters were employed to remove noises for real-time analysis. In this study, 6 electrodes Oz, O1, O2, P3, P4, and Pz are selected, which are located in the occipital areas while keeping their impedance values below 5 kΩ to record the original EEG signal. Reference (REF) and ground (GND) electrodes were used, respectively, as ground channel and reference channel. The bandpass filter with 7 Hz to 80 Hz and the 50 Hz notch filter were applied to remove artifacts of other physiological signals and powerline interference (PLI) (Zhang et al., 2012).

### 2.4. Experimental Design

Two types of experiments were implemented to access the performance of the OCT-CCA method and investigate the control capability of the SSVEP-based robotic arm system: (1) an offline comparison experiment for SSVEP recognition on an open SSVEP dataset. (2) online experiments for SSVEP recognition and real-time control of the 7-DOF manipulator to reach the specified position in 3D space.

#### 2.4.1. Experimental 1: An Offline Experiment for SSVEP Recognition Based on a Benchmark Dataset

A comparison experiment was employed on an open SSVEP dataset among the CCA, IT-CCA, and the OCT-CCA to explore the improvements of the proposed OCT-CCA algorithm. The benchmark dataset was adopted as it contains multiple subject data and numerous stimulus targets and has been used by many researchers to evaluate a new method, which collects EEG data from 35 subjects. For each subject, the SSVEP data was collected from 64 electrodes at the 40 stimulus frequencies (Wang et al., 2017). Here, the SSVEP data from subject 1 to subject 10 with 64 channels and 6 blocks are used for the offline experiment. The average recognition accuracy of stimulus targets was calculated by a leave-one-out cross-validation. The experimental results show the accuracy at different time window lengths (TW) from 1 to 5 s.

#### 2.4.2. Experimental 2: Online Experiments for Robotic Arm Movement Control in 3D Space

The real-time control experiments with five subjects were conducted for the 7-DOF robotic arm in a 3D space, and the specified movement tasks were implemented to estimate the feasibility of the system. Before this, the offline data was recorded to build OCTs, and a comparison experiment was completed with different time window lengths to ascertain a suitable time window length for the robotic arm control experiment. Subsequently, each subject can freely control the end effector of the robotic arm to move forward, backward, right, left, up, and down in 3D space to complete two designated tasks. During the real-time control experiments, when the system completes online SSVEP recognition, a red square will be displayed at the corresponding position of the recognition result, and then the robotic arm will act according to the translated command.

## 3. Results

### 3.1. Performance of SSVEP Recognition Based on a Benchmark Dataset

**Figure 4:**
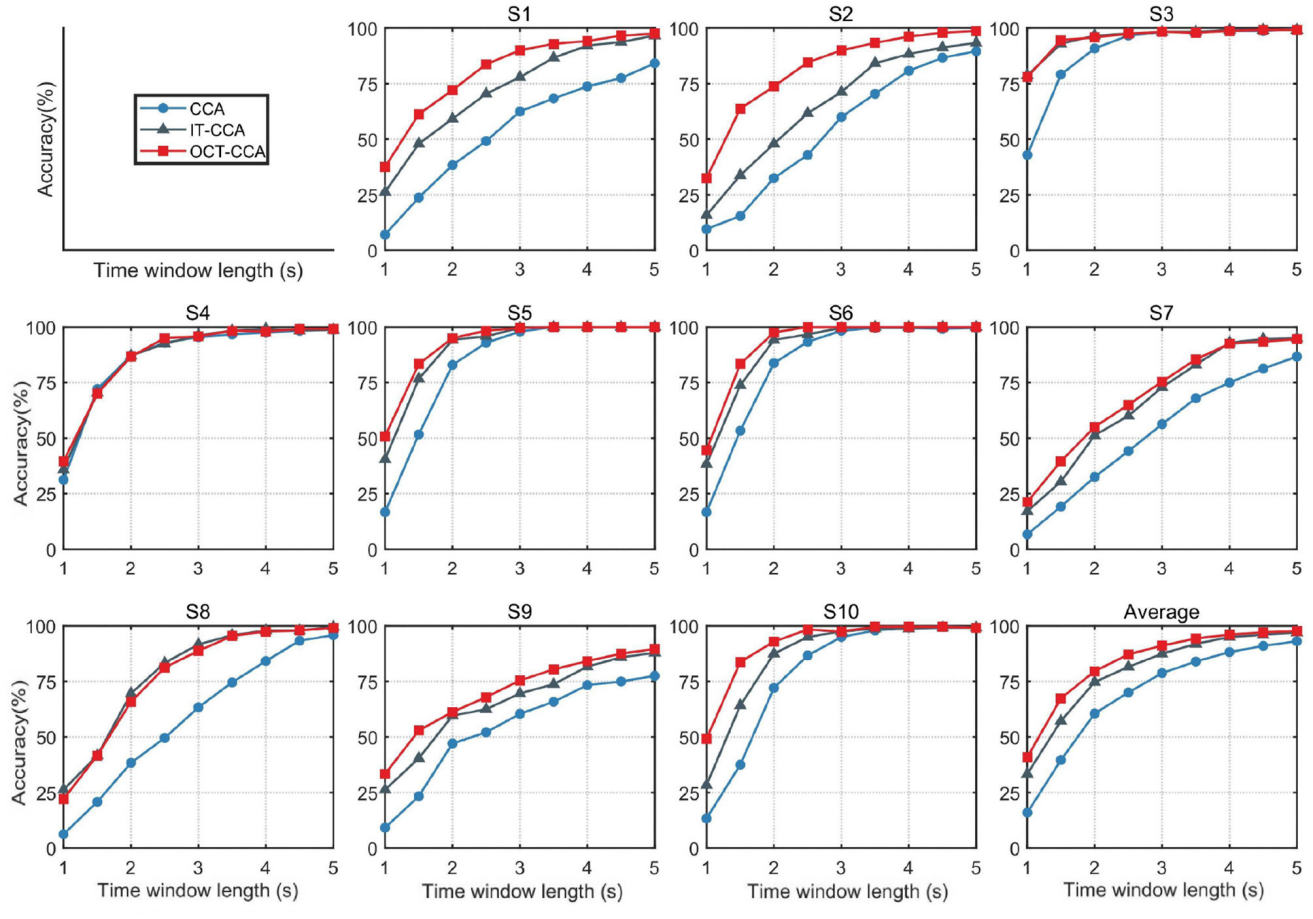
SSVEP recognition accuracy for the canonical correlation analysis (CCA), individual template-based canonical correlation analysis (IT-CCA), and OCT-CCA methods at time window lengths from 1 to 5 s with a step of 0.5 s.

Since the number of harmonics *N*_*h*_ will affect the recognition performance of the system in the CCA-based method, the higher harmonic frequencies can be used to improve the identification accuracy of EEG signals. In online experiments, the bandpass filter with 7 Hz to 80 Hz was applied to remove artifacts. In order to take full advantage of higher harmonics, the value of *N*_*h*_ was set to 5 uniformly, and all channels of the benchmark dataset were used for frequency recognition. Figure 4 shows the SSVEP recognition accuracy from subject1 to subject10 with different time window lengths from 1 to 5 s by using the CCA, IT-CCA, and OCT-CCA methods. The lower right corner of Figure 4 shows the average classification accuracy of subject1 to subject10 derived by the CCA, IT-CCA, and OCT-CCA methods. Obviously, for most subjects, both the proposed OCT-CCA and IT-CCA methods achieved better performance than CCA at all time window lengths. As the result shows, the average classification accuracy of the OCT-CCA method is higher than CCA and IT-CCA in every time window and a significant improvement is achieved when the time window was less than 3 s. To see more details of the results, Table 1 presents the significant difference on CCA vs. IT-CCA, OCT-CCA vs. CCA, and OCT-CCA vs. IT-CCA by applying the paired *t*-test. The calculations prove that the proposed OCT-CCA achieved outstanding average accuracy than CCA at all of the nine time windows (*p* < 0.001 at the TW is between 1 and 4.5 s, *p* < 0.01 at TW = 5 s), and also noticeably outperformed the IT-CCA at every time window (*p* < 0.001 at TW from 1 to 2.5 s, *p* < 0.01 at TW from 3 to 4 s, *p* < 0.05 at TW = 4.5 s and 5 s). Moreover, the improvement of stimulus frequencies detection between the methods illustrated above becomes more obvious with the reduction of time window lengths.

**Table 1:** Paired *t*-tests were used to compare significant differences in recognition accuracy among the canonical correlation analysis (CCA), individual template-based CCA (IT-CCA), and optimized common template-based CCA (OCT-CCA) methods.

**Method comparison**	**Time window**								
	**1.0s**	**1.5s**	**2.0s**	**2.5s**	**3.0s**	**3.5s**	**4.0s**	**4.5s**	**5.0s**
IT-CCA vs. CCA	***	***	***	***	***	***	***	***	**
OCT-CCA vs. CCA	***	***	***	***	***	***	***	***	**
OCT-CCA vs. IT-CCA	***	***	***	***	**	**	**	*	*

*p < 0.05, **p < 0.01, ***p < 0.001.

### 3.2. Performance of Robotic Arm Movement Control in 3D Space

**Figure 5:**
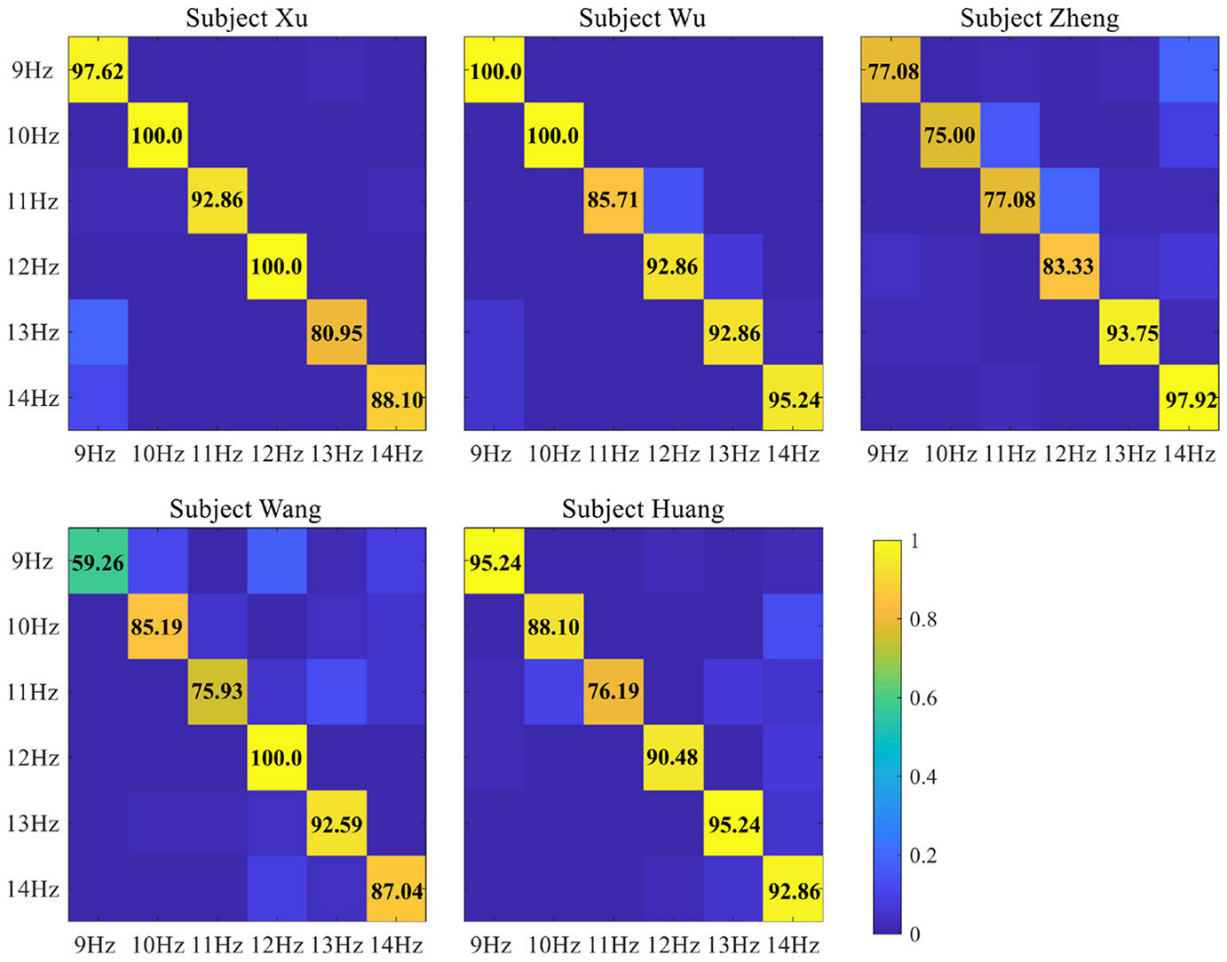
The recognition accuracy of each stimulus frequency at the time window of 2.5 s for five subjects.

In this section, the OCTs were bulit by recording EEG data from the five subjects. Then, the proposed OCT-CCA method is used for target detection at time window lengths from 1 to 5 s, which aims to fix a suitable time window length to complete the following robotic arm operations. In the subsystem of recognition, the proposed OCT-CCA was implemented to classify the six stimulus targets, and the classification accuracy of the five subjects for different time windows and the corresponding average accuracy was shown in Table 2. In general, the SSVEP recognition accuracy improved with the increase of time window lengths. From the results in the Table 2, it is known that within a time window length of 2 s, the average accuracy cross the five participants for the six-target identification was above 80%, and the average accuracy of 2.5 and 3.5 s were reached 90 and 95%, respectively. As described above, BCI also serves as a communication system, whose performance does not only relate to recognition accuracy, but also the information transfer rate (ITR) is another important performance parameter. Table 3 lists the simulated ITR across subjects using different time windows corresponding to the classification accuracy of Table 2. For all participants, the ITR reached the highest mean value at the 2.5 s time window for 45.99 ± 2.03 bits/min, which the maximum and minimum values were 47.47 bit/min (subject Xu, subject Wu and subject Huang) and 43.77 bit/min (subject Zheng and subject Wang), respectively, and the classification accuracy corresponding to the maximum ITR was 90.56 ± 1.52%. Considering the two factors of classification accuracy and ITR, it seems that 2.5 s is the most suitable time window. To further explore the feasibility of each stimulus frequency at the 2.5 s, the confusion matrices for classification accuracy of five subjects were illustrated in Figure 5, where the numbers on the diagonal indicate the accuracy for each target. Specifically, Subject Xu and Subject Wu achieved excellent performance in some individual targets, but Subject Zheng and Subject Wang showed unsatisfactory performance at some targets. The recognition accuracy of subject Zheng at the target frequency of 9 Hz, 10 Hz, and 11 Hz was less than 80%, even the accuracy of subject Wang was less than 60% at the target frequency of 9 Hz. Finally, we choose the time window of 3 s for the robotic arm to execute the task, where the recognition accuracy for each target frequency was above 80%.

**Table 2:** The OCT-CCA method was used to obtain the accuracy (%) of five subjects at time window lengths from 1 to 5 s.

**TW**	**Subject**					**Mean ±Std**
	**Xu**	**Wu**	**Zheng**	**Wang**	**Huang**	
1.0s	52.78	61.11	38.89	44.44	58.33	51.01 ± 9.50
1.5s	83.33	86.11	50.00	61.66	66.67	69.55 ± 15.14
2.0s	86.11	88.89	75.00	77.78	83.33	82.22 ± 5.76
2.5s	91.67	91.67	88.89	88.89	91.67	90.56 ± 1.52
3.0s	97.22	97.22	88.89	91.67	88.89	92.78 ± 4.21
3.5s	97.22	94.44	88.89	94.44	100.0	95.00 ± 4.12
4.0s	94.22	91.67	91.67	97.22	97.22	95.00 ± 3.04
4.5s	100.0	100.0	94.44	97.22	100.0	98.33 ± 2.49
5.0s	100.0	100.0	94.44	97.22	100.0	98.33 ± 2.49

**Table 3:** The OCT-CCA method was used to obtain the information transfer rate (ITR) (bits/min) of five subjects at different time window lengths.

**TW**	**Subject**					**Mean ±Std**
	**Xu**	**Wu**	**Zheng**	**Wang**	**Huang**	
1.0s	29.45	43.07	12.12	18.23	38.25	28.22 ± 13.05
1.5s	61.91	67.24	16.96	28.72	35.71	42.11 ± 21.66
2.0s	50.43	54.71	35.80	39.15	46.43	45.30 ± 7.81
2.5s	47.47	47.47	43.77	43.77	47.47	45.99 ± 2.03
3.0s	46.74	46.74	36.48	39.56	36.48	41.20 ± 5.21
3.5s	40.07	36.79	31.26	36.79	44.31	37.84 ± 4.80
4.0s	45.06	29.67	29.67	35.06	35.06	32.90 ± 2.95
4.5s	34.47	34.47	28.62	31.16	34.47	32.64 ± 2.66
5.0s	31.02	31.02	25.75	28.05	31.02	29.37 ± 2.40

**Figure 6:**
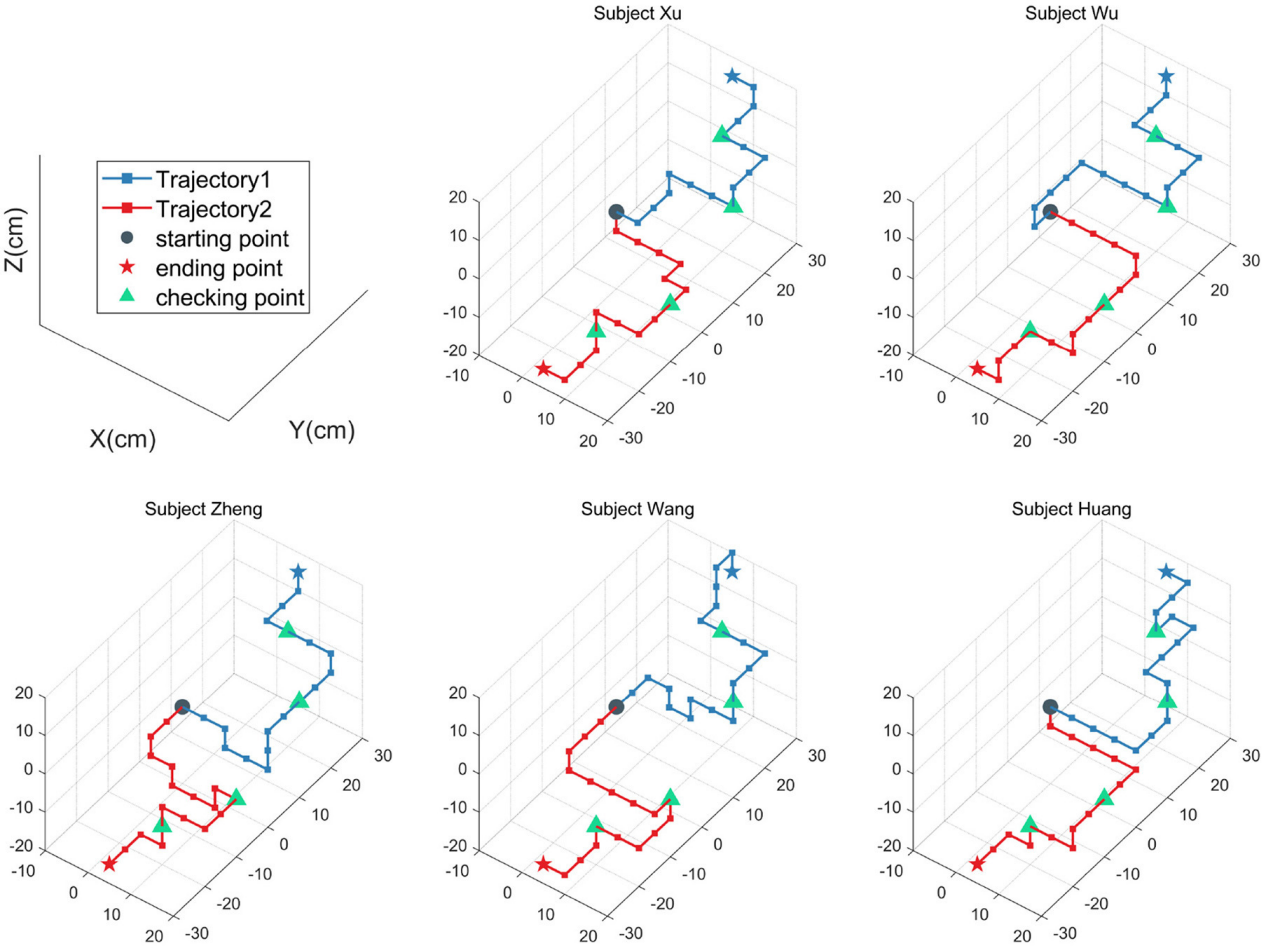
Trajectory path of the robotic arm in 3D space for each subject.

To visualize the online performance of movement control, each subject was required to fulfill two reaching tasks in 3D space without visual cues, and the trajectories of the robot arm's endpoint were recorded. Figure 6 shows the movement trajectories of the robotic arm to reach the designated target for five subjects, where each subject started from the same starting point and ended at the same ending point, different movement tasks and trajectories in the 3D space are distinguished by color. Specifically, the blue and red five-pointed stars indicate the ending point of task 1 and task 2, respectively, and the triangles indicate the checking points that need to be passed during the movement. As shown in Figure 6, all five subjects were capable to use the SSVEP-based BCI to control the manipulator to finish the two reaching tasks in 3D space successfully. The control commands also can be illustrated from the trajectories for five subjects. Table 4 lists the result of the reaching tasks for five subjects. As listed in Table 4, the five participants can finish tasks 1 and 2 by the average number of commands of 18 and 16, respectively, and the average total completion time was 174 ± 11.4 s. Since the SSVEP-based robotic arm control system in this paper is directly controlled by BCI, the subject can freely decide the next motion command of the robotic arm. Thereby, even though the five subjects have the same starting point and ending point, but showing different movement trajectories.

**Table 4:** Result of the reaching tasks of the robotic arm.

**Subject**	**Number of commands**		**Total completion time (s)**
	**Task 1**	**Task 2**	
Xu	16	16	160
Wu	18	16	170
Zheng	18	18	180
Wang	20	18	190
Huang	18	16	170
Mean ± Std	18 ± 1.41	16.8 ± 1.10	174 ± 11.40

## 4. Discussion and Conclusion

Nowadays, since higher ITR and low cost, the SSVEP-based BCIs are increasingly implemented in external device control fields such as robotic arm control. However, it is still challenging research to design an effective method to realize the control required for the complex tasks of a dexterous robotic arm, where the recognition accuracy is one of the most critical factors that cause the final control effect of the system. The CCA and its extended methods are the mainstream approaches for feature extraction in SSVEP-based BCI since their simple implementation and enhancing the SNR of SSVEP signals when using multiple channels (Wang et al., 2014). However, the reference signal of the CCA method is an overly idealized model, which is powerless to weaken the effect of spontaneous EEG and other background noise in the multi-channel signal due to the lack of real information (Wang et al., 2016; Zhang et al., 2020). At the same time, the template-based CCA method can optimize the reference signals by extracting more time-domain feature information from the EEG data. Thus, the template-based CCA methods significantly improved the performance and outperformed the training-free methods like FBCCA, and multivariate synchronization index (MSI) (Zerafa et al., 2018; Nakanishi et al., 2019).

In this study, the proposed OCT-CCA method was carried out to classify the six flicker targets of different stimulus frequencies, and then a non-invasive SSVEP-based BCI system was designed to control a robotic arm for reaching tasks in 3D space. In the proposed method, a common template was implemented to learn spatial filters used in correlation analysis for frequency recognition. The comparison result demonstrates that the templates constructed in the OCT-CCA method can extract more features within a shorter time window length, so it can achieve higher recognition accuracy than standard CCA and IT-CCA methods in the same time window lengths. This indicates that the OCT-CCA method is expected to improve the real-time computing control performance of the SSVEP-based BCI, which is also a key parameter that affects the performance of BCI to control other external devices. Furthermore, the outstanding classification ability of the improved method verifies that the common templates optimized from offline calibration data are more effective in extracting SSVEP-related components in EEG signals than the pre-constructed sine-cosine signals for SSVEP target recognition.

In the current study, the SSVEP-based BCI system was adopted instead of P300-based or motor imagery-based BCI is that the SSVEP paradigm can provide higher ITR and classifiable targets in real-time online systems P300, motor imagery, and other paradigms. In addition, when choosing the visual stimuli frequencies corresponding to the control commands, the low-frequencies (9–14 Hz, with a step of 1 Hz) stimuli were selected which can elicit strong SSVEP signals (Wittevrongel and Van Hulle, 2016). In this study, the 6-class SSVEP-based BCI system obtained the highest value of ITR of 45.99 bits/min and the corresponding average accuracy of 90.56% at 2.5 s time window length. To balance the recognition accuracy of each command to make the robotic arm work safely in the workspace, the final time window is determined to be 3 s. Therefore, it takes 5 s (3 s for gazing time, 2 s for robotic arm action, and gaze-shifting time) for the robotic arm control system to send out each command. To evaluate the effectiveness of the implemented robotic arm control system, all commands in six directions need to be recognized to complete the movement task in 3D space (shown in Figure 6). For future research, the hybrid paradigm is a new way that can improve the BCI performance by other bioelectric signals or external sensor signals sharing part of the pressure of EEG signal recognition. Where, for example, the depth camera is used to provide information about the external environment and instruct the robotic arm to move according to the command, while the subject only needs to select the target to be operated by the SSVEP-based BCI.

In summary, a novel OCT-based CCA method was proposed for target identification to perform the reaching tasks of a 7-DOF robotic arm in the 3D space. An offline experiment and online experiments were designed to confirm the improvements of the OCT-CCA method and the control performance of the robotic arm in the 3D space. The offline comparison results demonstrated that the classification accuracy of the OCT-CCA method outperforms the CCA and IT-CCA methods regardless of the time window lengths. The online experiment was completed by a controlled 7-DOF robotic arm, by focusing gaze on the flickering targets corresponding to the control command, subjects can manipulate the robotic arm as desired. The results showed that all five subjects can complete the designated reaching tasks within the appropriate time window. The success of the online experiment with five subjects demonstrates the simplicity and flexibility of the robotic arm control system in SSVEP-based BCI, which will be a practicable and promising application for the disabled in their daily lives.

## Data Availability Statement

Publicly available datasets were analyzed in this study. This data can be found here: Tsinghua University Brain-Computer Interface (BCI) Research Group, http://bci.med.tsinghua.edu.cn.

## Ethics Statement

The studies involving human participants were reviewed and approved by University of Electronic Science and Technology of China, Zhongshan Institute (Project identication code is 2021A0101180005). The patients/participants provided their written informed consent to participate in this study.

## Author Contributions

FP and ML explored the methodology, proposed the experiments, and analyzed the experimental data. ML and JX developed the BCI system and wrote the original draft. QX and HW developed the robotic arm control system and integrated the two systems. JX and HW performed the experiments and collected data. FP and S-nZ reviewed and edited the manuscript. All authors contributed to the article and approved the submitted version.

## Funding

This research is supposed in part by the National Natural Science Foundation of China under grant no. 62003312, in part by the Science and Technology Foundation of Guangdong Province under grant no. 2021A0101180005, in part by the Key Scientific Research Projects of Universities in Henan Province under grant no. 20A413011, in part by the Science and Technology Planning Project of Zhongshan under Grant 2019B2066.

## Conflict of Interest

The authors declare that the research was conducted in the absence of any commercial or financial relationships that could be construed as a potential conflict of interest.

## Publisher's Note

All claims expressed in this article are solely those of the authors and do not necessarily represent those of their affiliated organizations, or those of the publisher, the editors and the reviewers. Any product that may be evaluated in this article, or claim that may be made by its manufacturer, is not guaranteed or endorsed by the publisher.

## References

[B1] Aljalal M. Ibrahim S. Djemal R. Ko W. (2020). Comprehensive review on brain-controlled mobile robots and robotic arms based on electroencephalography signals. Intell. Service Rob. 13, 539–563. 10.1007/s11370-020-00328-5

[B2] Bin G. Gao X. Wang Y. Li Y. Hong B. Gao S. (2011). A high-speed bci based on code modulation vep. J. Neural Eng. 8, 025015. 10.1088/1741-2560/8/2/025015 21436527

[B3] Bonci A. Fiori S. Higashi H. Tanaka T. Verdini F. (2021). An introductory tutorial on brain-computer interfaces and their applications. Electronics 10, 560. 10.3390/electronics10050560

[B4] Borga M.. (1998). Learning multidimensional signal processing (Ph.D. thesis). Linköping University Electronic Press.

[B5] Chen S.-C. Chen Y.-J. Zaeni I. A. Wu C.-M. (2017). A single-channel ssvep-based bci with a fuzzy feature threshold algorithm in a maze game. Int. J. Fuzzy Syst. 19, 553–565. 10.1007/s40815-016-0289-3

[B6] Chen X. Huang X. Wang Y. Gao X. (2020). Combination of augmented reality based brain-computer interface and computer vision for high-level control of a robotic arm. IEEE Trans. Neural Syst. Rehabil. Eng. 28, 3140–3147. 10.1109/TNSRE.2020.3038209 33196442

[B7] Chen X. Wang Y. Gao S. Jung T.-P. Gao X. (2015). Filter bank canonical correlation analysis for implementing a high-speed ssvep-based brain-computer interface. J. Neural Eng. 12, 046008. 10.1088/1741-2560/12/4/046008 26035476

[B8] Chen X. Zhao B. Wang Y. Xu S. Gao X. (2018). Control of a 7-dof robotic arm system with an ssvep-based bci. Int. J. Neural Syst. 28, 1850018. 10.1142/S0129065718500181 29768990

[B9] Deng X. Yu Z. L. Lin C. Gu Z. Li Y. (2019). A bayesian shared control approach for wheelchair robot with brain machine interface. IEEE Trans. Neural Syst. Rehabil. Eng. 28, 328–338. 10.1109/TNSRE.2019.2958076 31825869

[B10] Farwell L. A. Richardson D. C. Richardson G. M. Furedy J. J. (2014). Brain fingerprinting classification concealed information test detects us navy military medical information with p300. Front. Neurosci. 8, 410. 10.3389/fnins.2014.00410 PMC4274905 25565941

[B11] Friman O. Cedefamn J. Lundberg P. Borga M. Knutsson H. (2001). Detection of neural activity in functional mri using canonical correlation analysis. Mag. Reson. Med. 45, 323–330. 10.1002/1522-2594(200102)45:2andlt;323::AID-MRM1041andgt;3.0.CO;2-# 11180440

[B12] Gao Q. Zhang Y. Wang Z. Dong E. Song X. Song Y. (2019). Channel projection-based cca target identification method for an ssvep-based bci system of quadrotor helicopter control. Comput. Intell. Neurosci. 2019, 2361282. 10.1155/2019/2361282 PMC6942778 31933620

[B13] Gao X. Xu D. Cheng M. Gao S. (2003). A bci-based environmental controller for the motion-disabled. IEEE Trans. Neural Syst. Rehabil. Eng. 11, 137–140. 10.1109/TNSRE.2003.814449 12899256

[B14] Ge S. Jiang Y. Wang P. Wang H. Zheng W. (2019). Training-free steady-state visual evoked potential brain-computer interface based on filter bank canonical correlation analysis and spatiotemporal beamforming decoding. IEEE Trans. Neural Syst. 1Rehabil. Eng. 27, 1714–1723. 10.1109/TNSRE.2019.2934496 31403435

[B15] Hakvoort G. Reuderink B. Obbink M. (2011). Comparison of psda and cca detection methods in a ssvep-based bci-system. Centre for Telematics and Information Technology University of Twente.

[B16] Hardoon D. R. Szedmak S. Shawe-Taylor J. (2004). Canonical correlation analysis: an overview with application to learning methods. Neural Comput. 16, 2639–2664. 10.1162/0899766042321814 15516276

[B17] Hwang H.-J. Lim J.-H. Jung Y.-J. Choi H. Lee S. W. Im C.-H. (2012). Development of an ssvep-based bci spelling system adopting a qwerty-style led keyboard. J. Neurosci. Methods 208, 59–65. 10.1016/j.jneumeth.2012.04.011 22580222

[B18] Kumar G. K. Reddy M. R. (2020). Constructing an exactly periodic subspace for enhancing ssvep based bci. Adv. Eng. Inf. 44, 101046. 10.1016/j.aei.2020.101046

[B19] Li Z. Zhao S. Duan J. Su C.-Y. Yang C. Zhao X. (2016). Human cooperative wheelchair with brain-machine interaction based on shared control strategy. IEEE/ASME Trans. Mechatron. 22, 185–195. 10.1109/TMECH.2016.2606642

[B20] Meng J. Zhang S. Bekyo A. Olsoe J. Baxter B. He B. (2016). Noninvasive electroencephalogram based control of a robotic arm for reach and grasp tasks. Sci. Rep. 6, 1–15. 10.1038/srep38565 27966546 PMC5155290

[B21] Nakanishi M. Wang Y. Chen X. Wang Y.-T. Gao X. Jung T.-P. (2017). Enhancing detection of ssveps for a high-speed brain speller using task-related component analysis. IEEE Trans. Biomed. Eng. 65, 104–112. 10.1109/TBME.2017.2694818 28436836 PMC5783827

[B22] Nakanishi M. Wang Y. Wang Y.-T. Jung T.-P. (2015). A comparison study of canonical correlation analysis based methods for detecting steady-state visual evoked potentials. PLoS ONE 10, e0140703. 10.1371/journal.pone.0140703 26479067 PMC4610694

[B23] Nakanishi M. Wang Y. Wang Y.-T. Mitsukura Y. Jung T.-P. (2014a). Enhancing unsupervised canonical correlation analysis-based frequency detection of ssveps by incorporating background EEG, in 2014 36th Annual International Conference of the IEEE Engineering in Medicine and Biology Society (Chicago, IL: IEEE), 3053–3056. 10.1109/EMBC.2014.6944267 25570635

[B24] Nakanishi M. Wang Y. Wang Y.-T. Mitsukura Y. Jung T.-P. (2014b). Generating visual flickers for eliciting robust steady-state visual evoked potentials at flexible frequencies using monitor refresh rate. PLoS ONE 9, e99235. 10.1371/journal.pone.0099235 24918435 PMC4053390

[B25] Nakanishi M. Wang Y.-T. Wei C.-S. Chiang K.-J. Jung T.-P. (2019). Facilitating calibration in high-speed bci spellers via leveraging cross-device shared latent responses. IEEE Trans. Biomed. Eng. 67, 1105–1113. 10.1109/TBME.2019.2929745 31329104

[B26] Nakanishi M. Wang Y. Jung T.-P. (2016). Session-to-session transfer in detecting steady-state visual evoked potentials with individual training data, in Foundations of Augmented Cognition: Neuroergonomics and Operational Neuroscience. AC 2016. Lecture Notes in Computer Science, eds D. Schmorrow and C. Fidopiastis (Cham: Springer), 9743. 10.1007/978-3-319-39955-3_24

[B27] Podmore J. J. Breckon T. P. Aznan N. K. Connolly J. D. (2019). On the relative contribution of deep convolutional neural networks for ssvep-based bio-signal decoding in bci speller applications. IEEE Trans. Neural Syst. Rehabil. Eng. 27, 611–618. 10.1109/TNSRE.2019.2904791 30872236

[B28] Rezeika A. Benda M. Stawicki P. Gembler F. Saboor A. Volosyak I. (2018). Brain-computer interface spellers: a review. Brain Sci. 8, 57. 10.3390/brainsci8040057 PMC5924393 29601538

[B29] Saidi P. Vosoughi A. Atia G. (2019). Detection of brain stimuli using ramanujan periodicity transforms. J. Neural Eng. 16, 036021. 10.1088/1741-2552/ab123a 30897556

[B30] Song M. Kim J. (2019). A paradigm to enhance motor imagery using rubber hand illusion induced by visuo-tactile stimulus. IEEE Transa. Neural Syst. Rehabil. Eng. 27, 477–486. 10.1109/TNSRE.2019.2895029 30703031

[B31] Sun L. Ji S. Ye J. (2010). Canonical correlation analysis for multilabel classification: a least-squares formulation, extensions, and analysis. IEEE Trans. Pattern Anal. Mach. Intell. 33, 194–200. 10.1109/TPAMI.2010.160 20733223

[B32] Wang H. Sun Y. Li Y. Chen S. Zhou W. (2020). Inter-and intra-subject template-based multivariate synchronization index using an adaptive threshold for ssvep-based bcis. Front. Neurosci. 14, 717. 10.3389/fnins.2020.00717 PMC7509063 33013279

[B33] Wang H. Zhang Y. Waytowich N. R. Krusienski D. J. Zhou G. Jin J.. (2016). Discriminative feature extraction via multivariate linear regression for ssvep-based bci. IEEE Transa. Neural Syst. Rehabil. Eng. 24, 532–541. 10.1109/TNSRE.2016.2519350 26812728

[B34] Wang Y. Chen X. Gao X. Gao S. (2017). A benchmark dataset for ssvep-based brain–computer interfaces. IEEE Transa. Neural Syst. Rehabil. Eng. 25, 1746–1752. 10.1109/TNSRE.2016.2627556 27849543

[B35] Wang Y. Nakanishi M. Wang Y.-T. Jung T.-P. (2014). Enhancing detection of steady-state visual evoked potentials using individual training data. Annu. Int. Conf. IEEE Eng. Med. Biol. Soc. 2014, 3037–3040. 10.1109/EMBC.2014.6944263 25570631

[B36] Wittevrongel B. Van Hulle M. M. (2016). Frequency-and phase encoded ssvep using spatiotemporal beamforming. PLoS ONE 11, e0159988. 10.1371/journal.pone.0159988 27486801 PMC4972379

[B37] Wong C. M. Wang Z. Wang B. Lao K. F. Rosa A. Xu P.. (2020). Inter-and intra-subject transfer reduces calibration effort for high-speed ssvep-based bcis. IEEE Transa. Neural Syst. Rehabil. Eng. 28, 2123–2135. 10.1109/TNSRE.2020.3019276 32841119

[B38] Xu Y. Ding C. Shu X. Gui K. Bezsudnova Y. Sheng X.. (2019). Shared control of a robotic arm using non-invasive brain-computer interface and computer vision guidance. Rob. Auton. Syst. 115, 121–129. 10.1016/j.robot.2019.02.014

[B39] Xu Y. Huang X. Lan Q. (2021). Selective cross-subject transfer learning based on riemannian tangent space for motor imagery brain-computer interface. Front. Neurosci. 1487, 779231. 10.3389/fnins.2021.779231 PMC8595943 34803600

[B40] Yin E. Zeyl T. Saab R. Hu D. Zhou Z. Chau T. (2016). An auditory-tactile visual saccade-independent p300 brain-computer interface. Int. J. Neural Syst. 26, 1650001. 10.1142/S0129065716500015 26678249

[B41] Yuan P. Chen X. Wang Y. Gao X. Gao S. (2015). Enhancing performances of ssvep-based brain-computer interfaces via exploiting inter-subject information. J. Neural Eng. 12, 046006. 10.1088/1741-2560/12/4/046006 26028259

[B42] Zerafa R. Camilleri T. Falzon O. Camilleri K. P. (2018). To train or not to train? a survey on training of feature extraction methods for ssvep-based bcis. J. Neural Eng. 15, 051001. 10.1088/1741-2552/aaca6e 29869996

[B43] Zhang Y. Xie S. Q. Wang H. Zhang Z. (2020). Data analytics in steady-state visual evoked potential-based brain-computer interface: a review. IEEE Sens. J. 21, 1124–1138. 10.1109/JSEN.2020.3017491

[B44] Zhang Y. Xu P. Liu T. Hu J. Zhang R. Yao D. (2012). Multiple frequencies sequential coding for ssvep-based brain-computer interface. PLoS ONE 7, e29519. 10.1371/journal.pone.0029519 22412829 PMC3295792

[B45] Zhang Y. Zhou G. Jin J. Wang M. Wang X. Cichocki A. (2013). L1-regularized multiway canonical correlation analysis for ssvep-based bci. IEEE Transa. Neural Syst. Rehabil. Eng. 21, 887–896. 10.1109/TNSRE.2013.2279680 24122565

[B46] Zhang Y. Zhou G. Zhao Q. Onishi A. Jin J. Wang X.. (2011). Multiway canonical correlation analysis for frequency components recognition in ssvep-based bcis, in Neural Information Processing. ICONIP 2011. Lecture Notes in Computer Science, eds B. L. Lu, L. Zhang, and J. Kwok (Berlin; Heidelberg: Springer), 7062. 10.1007/978-3-642-24955-6_35

[B47] Zhu Y. Li Y. Lu J. Li P. (2020). A hybrid bci based on ssvep and eog for robotic arm control. Front. Neurorobot. 14, 95. 10.3389/fnbot.2020.583641 PMC7714925 33328950

